# Nutritional Regulation of Reproductive Physiology in Ruminants: A Mechanistic Review

**DOI:** 10.3390/life16040630

**Published:** 2026-04-08

**Authors:** Ting-Chieh Kang, Geng-Jen Fan, Hisn-Hung Lin, Kai-Fei Tseng, Ya-Chun Liu, Hsi-Hsun Wu

**Affiliations:** 1 Southern Region Branch, Livestock Research Institute, Ministry of Agriculture, Pingtung 912, Taiwan; hhlin@tlri.gov.tw (H.-H.L.); kfsteng@tlri.gov.tw (K.-F.T.); ycliu@tlri.gov.tw (Y.-C.L.); 2 Taiwan Livestock Research Institute, Ministry of Agriculture, Tainan 712, Taiwan; m38208@mail.tlri.gov.tw; 3 Department of Animal Science, National Chiayi University, Chiayi 621, Taiwan

**Keywords:** ruminant reproduction, nutritional physiology, cattle, sheep, negative energy balance, metabolic hormones, kisspeptin, fetal programming, antioxidants, precision feeding

## Abstract

Modern genetic selection for high productivity has created a physiological conflict in ruminants, where the metabolic demands of lactation compete directly with the energy requirements of reproduction. This review provides a mechanistic synthesis of how key nutritional factors modulate the endocrine and cellular pathways governing reproductive success in cattle and sheep. Negative energy balance (NEB), characteristic of the early postpartum period, suppresses the hypothalamic–pituitary–gonadal (HPG) axis by impairing the pulsatile secretion of gonadotropin-releasing hormone (GnRH) and luteinizing hormone (LH), mediated through reduced kisspeptin signaling, growth hormone (GH) resistance, and decreased circulating insulin, insulin-like growth factor-1 (IGF-1), and leptin. At the macronutrient level, excess rumen-degradable protein elevates blood urea nitrogen and impairs the uterine environment, while omega-3 polyunsaturated fatty acids inhibit prostaglandin F2α synthesis to support corpus luteum maintenance. At the micronutrient level, selenium, copper, and zinc are essential antioxidant cofactors protecting gametes and embryos from oxidative stress, while vitamins A, D, and E regulate gene expression in reproductive tissues. Furthermore, maternal nutrition during critical gestational windows programs the reproductive capacity of offspring through epigenetic modifications, with profound implications for long-term herd fertility. Understanding these nutritional–reproductive interactions is crucial for developing precision feeding strategies that optimize herd fertility, improve animal welfare, and ensure the economic sustainability of livestock management. A thorough understanding of these nutritional–reproductive interactions is essential for developing precision feeding strategies that optimize fertility in high-producing ruminants.

## 1. Introduction

Modern genetic selection for high productivity in ruminants has created a significant physiological challenge, where the metabolic drive for milk or meat production directly competes with the energy-intensive processes of reproduction. This antagonism is a primary factor contributing to the well-documented decline in fertility observed in high-yielding dairy cattle [1,2] and prolific sheep breeds [3,4]. While the fundamental physiological mechanisms discussed herein apply to all ruminants, specific metabolic challenges (e.g., severe negative energy balance) are primarily addressed in the context of dairy cattle, whereas grazing nutrition is discussed with reference to beef cattle and sheep. The postpartum period is particularly critical, as animals enter a state of negative energy balance (NEB), where energy expenditure exceeds energy intake. During NEB, physiological priority is given to lactation and the maintenance of vital functions, while reproductive activity is suppressed [5]. Nutrition serves as a critical regulator of this complex interplay, acting not merely as a source of metabolic fuel but as a provider of potent signaling molecules that modulate the endocrine pathways governing reproduction. The availability of energy, proteins, fatty acids, vitamins, and minerals directly influences the function of the hypothalamic–pituitary–gonadal (HPG) axis, ovarian folliculogenesis, oocyte quality, and the uterine environment [6]. An inadequate or imbalanced nutritional status can delay the resumption of postpartum ovarian cyclicity, reduce conception rates, and increase the incidence of early embryonic loss. This review provides a mechanistic overview of the nutritional regulation of reproductive physiology in cattle and sheep. We examine how systemic energy status and specific dietary components are integrated at the molecular and endocrine levels to control key reproductive events, from gametogenesis to the establishment of pregnancy. A thorough understanding of these mechanisms is essential for developing targeted nutritional strategies to mitigate the conflict between production and fertility, thereby improving the overall efficiency and sustainability of ruminant production systems.

## 2. The Role of Energy Balance in Reproductive Function

**Figure 1:**
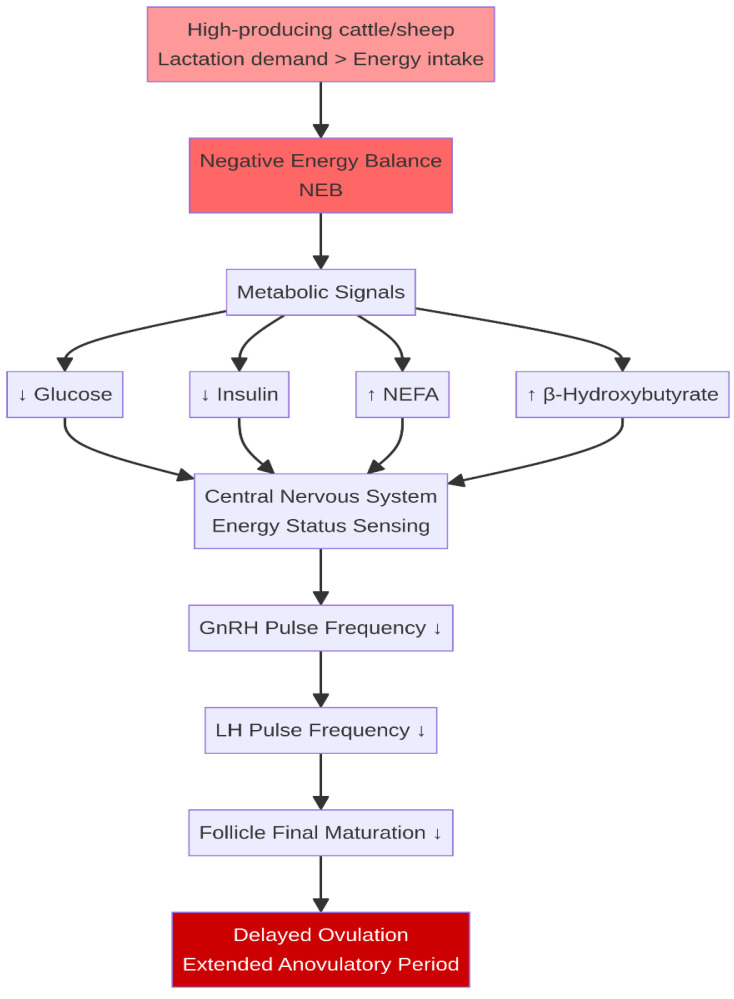
Mechanism of negative energy balance (NEB) inhibition of the hypothalamic–pituitary–gonadal (HPG) axis. High lactation demands lead to NEB, characterized by metabolic signals (↓ Glucose, ↓ Insulin, ↑ NEFA, ↑ BHB) that suppress GnRH and LH pulsatility via reduced kisspeptin signaling, resulting in delayed ovulation. (Diagram created by the authors, synthesizing concepts from multiple cited studies). ↑ indicates an increase; ↓ indicates a decrease.

Energy balance is the most critical determinant of reproductive resumption in postpartum ruminants. The onset and duration of NEB are directly correlated with the length of the postpartum anovulatory interval [5]. High-producing dairy breeds (e.g., Holstein-Friesian) experience significantly more severe NEB compared to dual-purpose or beef breeds, leading to a longer delay in the resumption of ovarian activity [7]. Specifically, prepartum non-esterified fatty acid (NEFA) concentrations >0.4 mmol/L and postpartum beta-hydroxybutyrate (BHB) concentrations >1.2 mmol/L are strongly associated with delayed first ovulation and reduced conception rates [8,9]. The metabolic state of NEB is characterized by low circulating concentrations of glucose and insulin, coupled with elevated levels of NEFA and BHB mobilized from adipose tissue [6]. These metabolic cues are sensed by the central nervous system and directly impact the pulsatile secretion of GnRH from the hypothalamus, which in turn suppresses the frequency of LH and follicle-stimulating hormone (FSH) pulses from the pituitary gland [5,6]. Since LH pulsatility is required to drive the final maturation and ovulation of dominant follicles, its suppression is the primary cause of anovulation during NEB (Figure 1).

Beyond its central effects, the metabolic milieu of NEB has direct consequences at the ovarian level. Elevated concentrations of NEFA in the follicular fluid induce lipotoxicity, causing apoptosis in granulosa cells and impairing steroidogenesis. This directly compromises the developmental competence of the oocyte, ultimately leading to reduced blastocyst formation rates and early embryonic loss [10,11,12]. Additionally, ruminants uniquely rely on gluconeogenesis and experience massive adipose tissue mobilization during early lactation, exacerbating these lipotoxic effects [8]. This creates a suboptimal follicular environment that can compromise the viability of the resulting embryo, even if fertilization occurs.

The body condition score (BCS) serves as a practical indicator of an animal’s energy reserves. An optimal BCS (e.g., 3.0–3.5 on a 5-point scale for dairy cows) at parturition is associated with a shorter anovulatory period and improved conception rates, as it provides the necessary metabolic buffer to mitigate the severity of NEB [13]. Conversely, both excessively low and high BCS are linked to diminished fertility, due to insufficient energy reserves or severe metabolic dysfunction associated with excessive fat mobilization, respectively [14].

In sheep, the practice of “flushing” (a short-term increase in dietary energy supply prior to mating) effectively demonstrates the sensitivity of the HPG axis to acute changes in energy status. Flushing increases circulating glucose and insulin, which stimulates LH pulsatility and leads to an increased ovulation rate [15,16]. This highlights the direct link between nutrient availability and the modulation of ovarian function.

## 3. Macronutrient Modulation of the Reproductive Axis

Beyond overall energy status, the specific composition of macronutrients, particularly proteins and fatty acids, exerts significant modulatory effects on reproductive physiology. Furthermore, the rumen microbiome plays a critical role in metabolizing dietary components, such as converting plant polyphenols into phytoestrogens (e.g., equol) that can disrupt the estrous cycle [17].

### 3.1. Protein and Amino Acids

**Figure 2:**
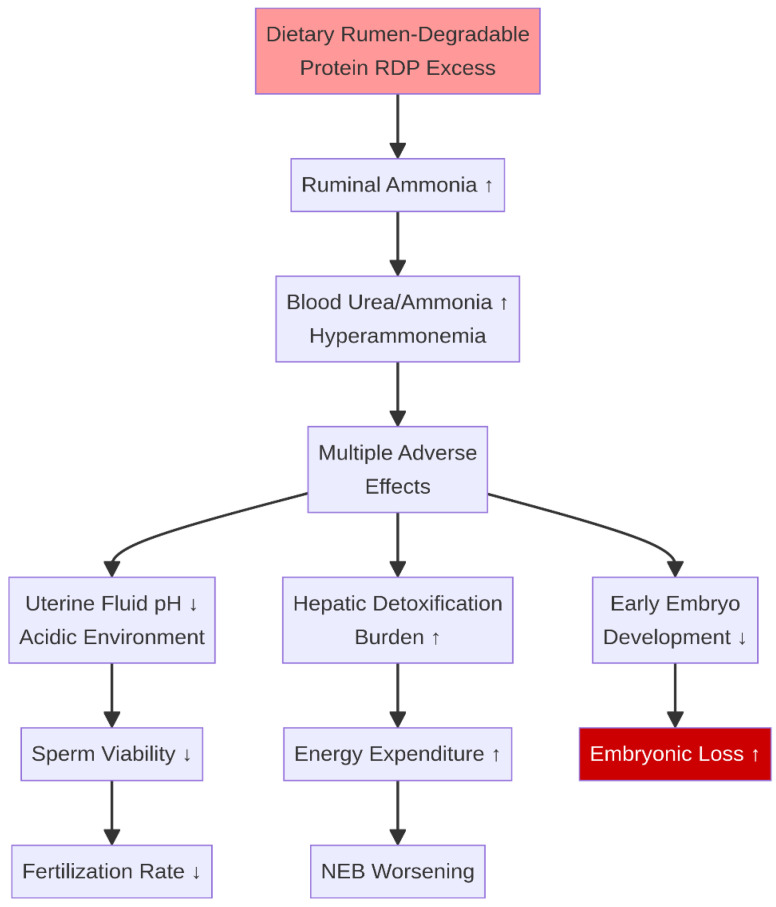
Harmful effects of excess dietary protein. Excessive rumen-degradable protein (RDP) leads to hyperammonemia, causing an acidic uterine environment, increased hepatic detoxification burden, and impaired embryo development. ↑ indicates an increase; ↓ indicates a decrease.

Dietary protein intake must be carefully balanced, as both deficiency and excess can negatively impact fertility. An excess of rumen-degradable protein (RDP) relative to the energy available for microbial protein synthesis leads to elevated concentrations of ammonia and urea in the blood and uterine fluids [18]. Dietary RDP exceeding 10% of dry matter (DM) intake is associated with elevated blood urea nitrogen (BUN) and milk urea nitrogen (MUN) levels, which can impair the uterine environment and reduce conception rates [19,20,21]. This hyperammonemia can alter the pH of the uterine luminal environment, creating conditions that are suboptimal for sperm viability, fertilization, and early embryonic development [22] (Figure 2). Furthermore, the energetic cost of converting excess ammonia to urea in the liver places an additional metabolic burden on the animal, exacerbating the effects of NEB [23].

**Figure 3:**
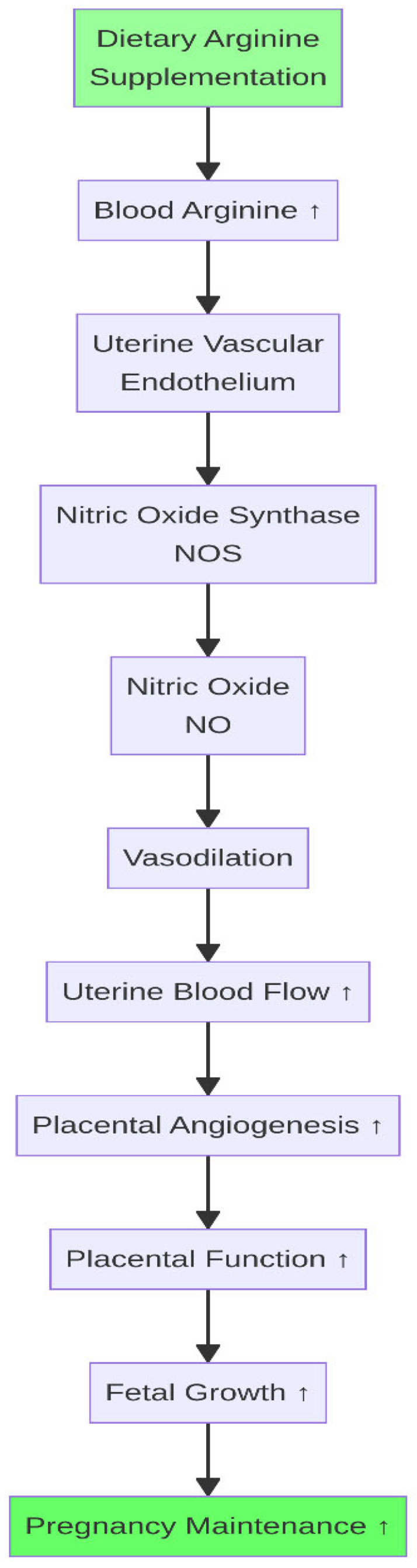
Arginine–nitric oxide (NO) pathway. Dietary arginine supplementation increases blood arginine, which is converted by nitric oxide synthase (NOS) to NO, promoting vasodilation, uterine blood flow, and placental angiogenesis. ↑ indicates an increase.

In contrast, supplementing specific rumen-protected amino acids has shown considerable promise. Methionine is a precursor for key antioxidants such as glutathione and plays a role in hepatic lipid metabolism and one-carbon metabolic pathways; its supplementation during the transition period has been associated with improved immune function, reduced hepatic lipid accumulation, and better postpartum fertility outcomes [24,25]. Arginine is the substrate for nitric oxide (NO) synthesis by nitric oxide synthase (NOS). NO is a potent vasodilator that is crucial for placental angiogenesis and uterine blood flow, supporting both early embryonic development and conceptus elongation [26,27,28] (Figure 3).

### 3.2. Fatty Acids

**Figure 4:**
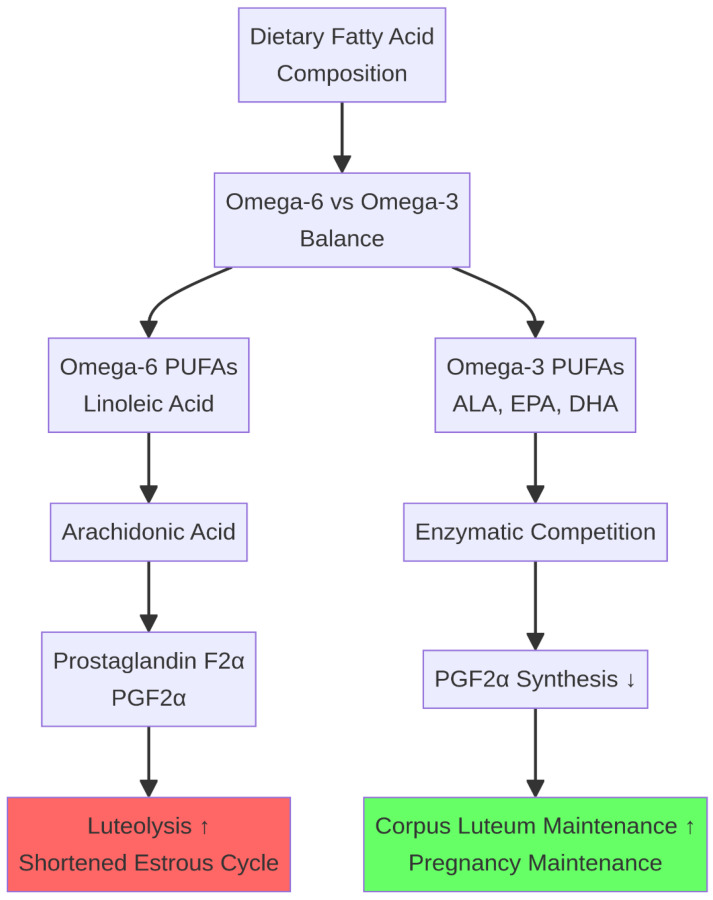
Fatty acid regulation of corpus luteum function. Omega-6 PUFAs promote PGF2α synthesis, leading to luteolysis, while Omega-3 PUFAs inhibit PGF2α synthesis, promoting corpus luteum maintenance and supporting maternal recognition of pregnancy. ↑ indicates an increase; ↓ indicates a decrease.

Dietary fatty acids are not only a concentrated energy source but also potent signaling molecules that modulate reproductive processes, primarily through their role as precursors for eicosanoids. The balance between omega-6 and omega-3 polyunsaturated fatty acids (PUFAs) is of particular importance. Omega-6 PUFAs, such as linoleic acid, are precursors to arachidonic acid and subsequently prostaglandin F2α (PGF2α), the primary luteolytic agent in ruminants [29]. An excessive dietary intake of omega-6 PUFAs can promote premature luteolysis and shorten the estrous cycle. Conversely, omega-3 PUFAs, such as α-linolenic acid (ALA), eicosapentaenoic acid (EPA), and docosahexaenoic acid (DHA), compete with omega-6 PUFAs for enzymatic conversion and can inhibit the synthesis of PGF2α [30]. This anti-luteolytic effect helps to ensure the maintenance of the corpus luteum (CL) and is critical for the maternal recognition of pregnancy. While many studies report that omega-3 supplementation improves embryo survival and luteal function, results can be variable depending on the basal diet and physiological state [31]. Practical dietary sources commonly used in ruminant diets include flaxseed, fish oil, and rumen-protected marine microalgae, typically supplemented at 1–3% of dry matter, which have been associated with reduced pregnancy loss in dairy cattle [32,33,34] (Figure 4).

## 4. Micronutrient Regulation of Reproductive Processes

Micronutrients, including trace minerals and vitamins, function as essential cofactors for enzymes and as antioxidants, playing indispensable roles in reproductive health. Table 1 summarizes the primary reproductive functions, deficiency consequences, and recommended supply ranges for key micronutrients in ruminants (Based on National Academies of Sciences, Engineering, and Medicine (NASEM)/NRC guidelines). Note that specific requirements and maximum tolerable levels (e.g., Copper) differ between cattle and sheep [35].

### 4.1. Trace Minerals and Antioxidant Defense

**Figure 5:**
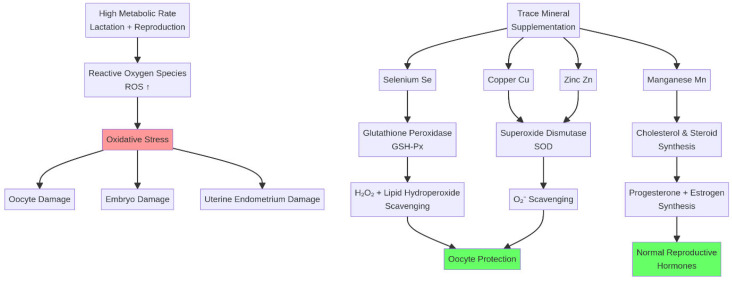
Trace mineral antioxidant defense system. Trace minerals (Se, Cu, Zn, Mn) are essential cofactors for antioxidant enzymes (GSH-Px, SOD) that neutralize reactive oxygen species (ROS), protecting gametes and embryos from oxidative stress. ↑ indicates an increase.

The high metabolic rate associated with lactation and reproduction leads to increased production of reactive oxygen species (ROS), resulting in oxidative stress. Oxidative stress can inflict significant damage on gametes and embryos, which are particularly vulnerable due to their high lipid content and limited antioxidant capacity [39]. Several trace minerals are critical components of the endogenous antioxidant system. Selenium (Se) is a key constituent of the enzyme glutathione peroxidase (GSH-Px), which neutralizes hydrogen peroxide and lipid hydroperoxides [36,37]. Copper (Cu) and Zinc (Zn) are essential cofactors for the enzyme superoxide dismutase (SOD), which catalyzes the dismutation of the superoxide radical. Manganese (Mn) similarly serves as a cofactor for mitochondrial SOD and is required for cholesterol and steroid synthesis. Deficiencies in these minerals compromise the animal’s ability to counteract oxidative stress, leading to increased incidence of reproductive disorders such as retained fetal membranes, metritis, and early embryonic death [40,41] (Figure 5).

Furthermore, these minerals have direct, synergistic roles in supporting reproductive endocrinology and mitigating oxidative stress, which are critical for steroidogenesis, GnRH regulation, and maintaining gamete integrity [35,36,42,43]. Cobalt (Co), as the central atom of vitamin B12 (cobalamin), is required for methionine synthase activity and the maintenance of one-carbon metabolic pathways critical for fetal development and placental function [44].

### 4.2. Vitamins as Signaling Molecules

**Figure 6:**
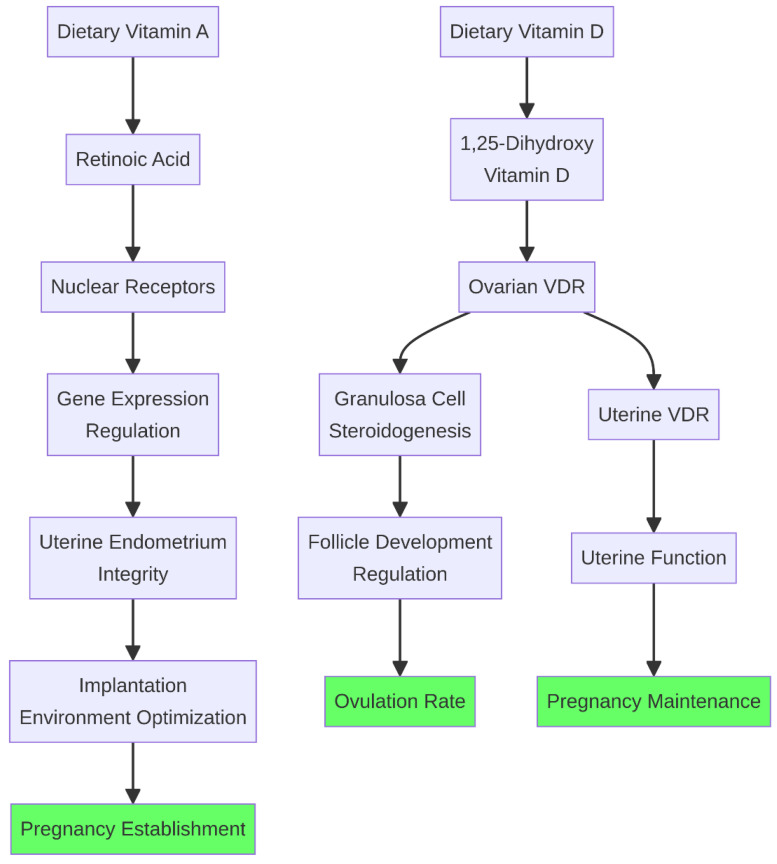
Vitamin A, D, and E signal transduction in reproductive tissues. Vitamin A (as retinoic acid) and Vitamin D regulate gene expression through nuclear receptors (RAR/RXR and VDR, respectively), influencing uterine integrity and follicle development. Vitamin E acts as a membrane-protective antioxidant in synergy with selenium.

Fat-soluble vitamins, particularly vitamins A, D, and E, function beyond their traditional roles and act as hormone-like signaling molecules that regulate gene expression and cellular integrity in reproductive tissues (Figure 6).

Vitamin A and its primary active metabolite, retinoic acid, are essential for maintaining the integrity of epithelial tissues, including the uterine endometrium. Retinoic acid binds to nuclear receptors (RAR/RXR) and modulates the expression of genes involved in cellular proliferation and differentiation, which are critical for implantation and placentation [45]. Its precursor, β-carotene, has been shown to accumulate in the corpus luteum and follicular fluid of cattle, where it may play a direct antioxidant role and support progesterone synthesis [46]. Supplementation with β-carotene has been associated with improved conception rates and reduced embryonic mortality in periparturient dairy cows [47].

Vitamin D receptors (VDR) have been identified in the ovary, uterus, and pituitary gland of ruminants. Vitamin D has been shown to influence ovarian steroidogenesis in granulosa cells and may play a role in regulating follicular development and immune tolerance at the fetal-maternal interface [48,49]. In a free-ranging sheep population, higher vitamin D status was associated with greater reproductive fitness, underscoring the physiological relevance of this pathway under natural conditions [49].

Vitamin E (α-tocopherol) functions synergistically with selenium to form a critical antioxidant network. It is the principal lipid-soluble antioxidant protecting cell membranes, including those of gametes and uterine cells, from peroxidative damage. The peripartum period is characterized by a dramatic decline in circulating vitamin E, coinciding with increased oxidative stress. Parenteral supplementation of vitamin E with selenium in transition cows has been associated with significant reductions in the incidence of retained fetal membranes, metritis, and other periparturient disorders that impair subsequent fertility [40,47].

## 5. Integration by Metabolic Hormones: The Endocrine Interface

**Figure 7:**
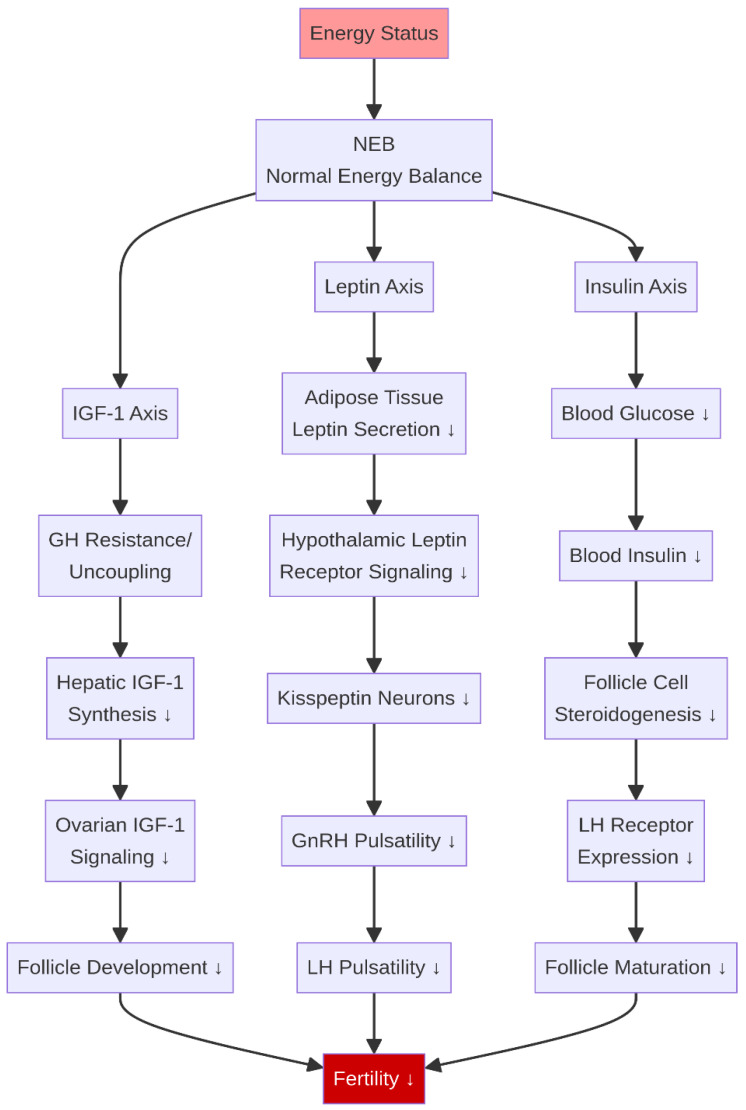
Metabolic hormone integration: the energy–reproduction interface. During NEB, suppression of the IGF-1, leptin, and insulin axes, and the consequent reduction in kisspeptin (KISS1) signaling, converges to reduce fertility by impairing follicle development and GnRH/LH pulsatility. ↓ indicates a decrease.

The nutritional status of an animal is communicated to the reproductive axis through a complex network of metabolic hormones that act as an interface between metabolism and fertility (Figure 7). Key hormones in this network include insulin-like growth factor 1 (IGF-1), leptin, and insulin.

### 5.1. The GH–IGF-1 Axis

The growth hormone (GH)–IGF-1 axis is profoundly affected by NEB. During this state, a phenomenon known as GH resistance or hepatic uncoupling occurs, where the liver becomes refractory to GH stimulation despite elevated circulating GH levels, leading to a significant reduction in IGF-1 synthesis and secretion [50]. This is a key metabolic adaptation to partition nutrients toward milk production, a trait inadvertently amplified by genetic selection for high yields [51]. IGF-1 is a critical permissive factor for ovarian function; it acts synergistically with gonadotropins to stimulate granulosa cell proliferation, steroidogenesis, and oocyte maturation. Consequently, the low IGF-1 environment during NEB impairs follicular development and reduces fertility [51,52].

### 5.2. Leptin and the Kisspeptin–GnRH Axis

Leptin, a hormone primarily secreted by adipose tissue, serves as a long-term indicator of the body’s energy reserves, and its circulating concentrations decrease dramatically during NEB. Leptin receptors are present in the hypothalamus, pituitary, and ovary, indicating its widespread role in reproductive regulation. A primary mechanism of leptin action on reproduction is through the stimulation of hypothalamic neurons that produce kisspeptin (encoded by the KISS1 gene), which is the most potent known secretagogue of GnRH. Kisspeptin-expressing neurons in the arcuate nucleus (ARC), often co-expressing Neurokinin B and Dynorphin—collectively termed kisspeptin-neurokinin B-dynorphin (KNDy) neurons—form the core pulse generator of the GnRH system. Low leptin levels during NEB lead to reduced kisspeptin signaling from KNDy neurons, which is a primary contributing mechanism to the suppression of GnRH and LH pulsatility observed in energy-deficient animals [53,54]. While the detailed molecular interactions of the leptin-kisspeptin pathway are partly extrapolated from rodent models, recent evidence confirms the functional role of kisspeptin and its receptors in the ruminant hypothalamus and gonads [55]. Recent transcriptomic and metabolomic studies have further elucidated the molecular regulatory networks of the hypothalamus and ovaries during sexual maturation and nutritional stress in ruminants [56,57]. Studies in undernourished ewes have demonstrated that central administration of leptin can partially restore LH secretion, confirming its crucial role as a metabolic gatekeeper for reproduction [58].

### 5.3. Insulin

Insulin, a key regulator of glucose metabolism, also functions as a metabolic signal to the reproductive system. The hypoinsulinemia characteristic of NEB contributes to the suppression of GnRH secretion and reduces the responsiveness of the ovary to LH. Insulin directly stimulates steroidogenesis in both theca and granulosa cells and enhances the expression of LH receptors on granulosa cells, thereby promoting follicular growth and maturation [59]. The collective suppression of IGF-1, leptin, and insulin during NEB creates a powerful endocrine signal that places the reproductive system in a quiescent state until a more favorable metabolic environment is restored.

## 6. Male Reproductive Function and Nutritional Status

While much of the literature has focused on the female, nutritional status also profoundly influences male reproductive function in ruminants. The nutritional regulation of the HPG axis in males mirrors that in females; NEB and micronutrient deficiencies impair testosterone synthesis, spermatogenesis, and sperm quality, with significant consequences for overall herd fertility.

Adequate energy and protein supply are prerequisites for normal testicular function. Restriction of dietary energy reduces circulating LH and testosterone concentrations, leading to reduced libido and impaired spermatogenesis [60]. Oxidative stress damages sperm DNA and reduces motility. Supplementing organic trace minerals (zinc and copper) and antioxidants enhances antioxidant enzyme activity (e.g., SOD, catalase), improving semen volume and motility [61,62]. Specific micronutrients are critically important for sperm integrity: zinc is essential for the structural stability of sperm chromatin and the acrosomal reaction, while selenium is required for the formation of phospholipid hydroperoxide glutathione peroxidase (PHGPx/GPx5), a selenoprotein incorporated into the sperm midpiece that protects against oxidative damage to sperm membranes and mitochondria [37,38]. Copper deficiency impairs testosterone biosynthesis, and manganese deficiency has been associated with testicular degeneration. The dietary ratio of omega-6 to omega-3 PUFAs also influences sperm membrane fatty acid composition, which directly affects sperm membrane fluidity and fertilizing capacity; diets enriched in long-chain omega-3 PUFAs (EPA and DHA) have been shown to improve sperm quality parameters in rams and bulls [30]. Furthermore, PUFAs, particularly DHA, are critical for maintaining sperm membrane fluidity and cryotolerance [63].

## 7. Fetal Programming and Intergenerational Effects

**Figure 8:**
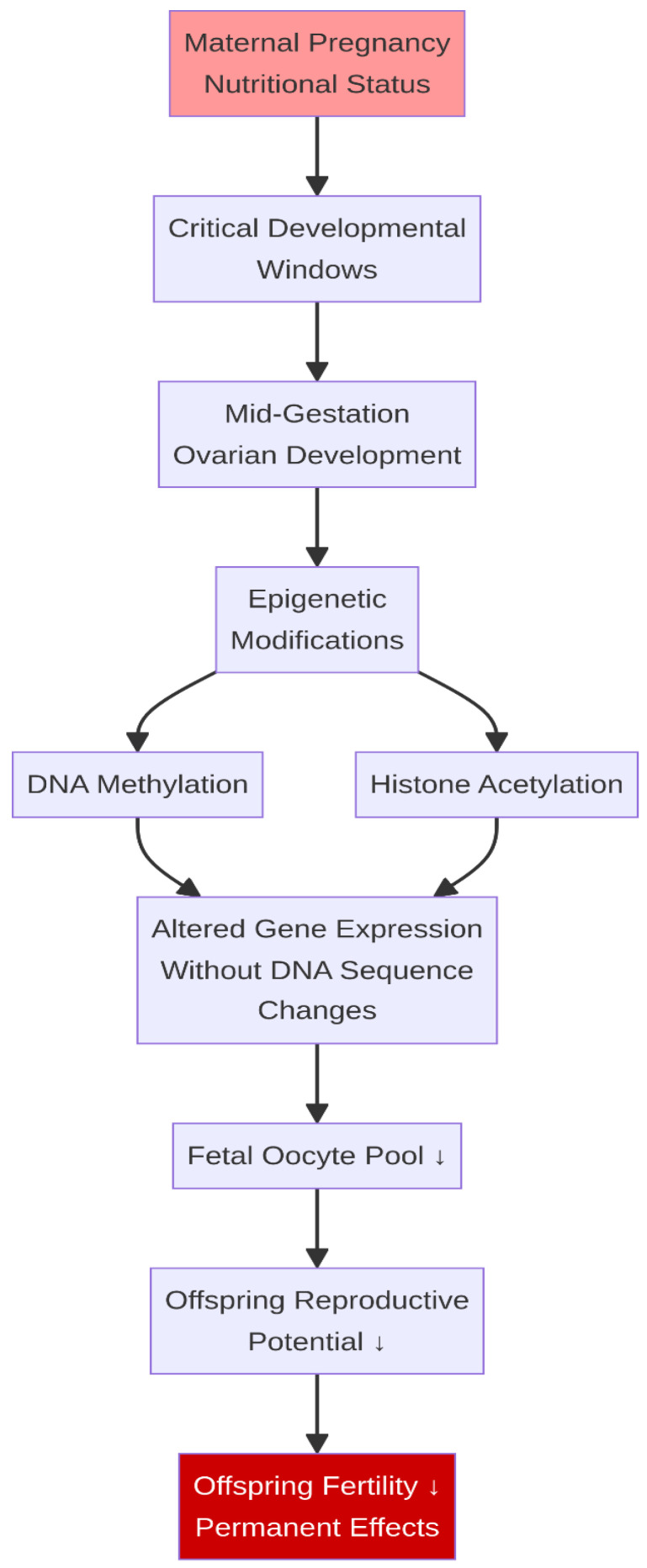
Fetal programming: transgenerational effects of maternal nutrition. Maternal nutritional status during critical developmental windows induces epigenetic modifications (DNA methylation, histone acetylation) that permanently alter gene expression, reducing the offspring’s reproductive potential across multiple organ systems. ↓ indicates a decrease.

The influence of nutrition on reproduction extends beyond the immediate dam to her offspring through the process of fetal programming. Maternal nutritional status during critical windows of gestation can permanently alter the development and function of the offspring’s reproductive system [64]. For example, maternal undernutrition during mid-gestation, when ovarian follicular development is initiated in the female fetus, can result in a reduced primordial follicle pool, thereby limiting the offspring’s lifetime reproductive potential [65]. These effects are mediated by epigenetic modifications, such as DNA methylation and histone acetylation, which alter the expression of genes controlling reproductive development and function without changing the underlying DNA sequence [57,66,67] (Figure 8).

Similarly, maternal nutrition can program the development of the male reproductive tract. Inadequate nutrient supply during gestation has been shown to reduce testicular size, Sertoli cell number, and daily sperm production in male offspring, with lifelong consequences for fertility [68]. Conversely, maternal overnutrition and obesity during gestation can also exert detrimental programming effects, including dysregulation of hypothalamic appetite and reproductive centers in the offspring, and altered fatty acid metabolism in fetal adipose tissue [69]. Both maternal undernutrition and overnutrition can lead to altered epigenetic regulation, resulting in metabolic disorders and compromised reproductive development (e.g., altered ovarian follicular reserves) in the offspring, thereby affecting long-term herd productivity [70,71]. These findings highlight the profound and bidirectional intergenerational impact of nutritional management, underscoring the importance of optimizing the nutritional status of the dam not only for her own fertility but for the reproductive capacity of future generations.

## 8. Conclusions and Future Directions

**Figure 9:**
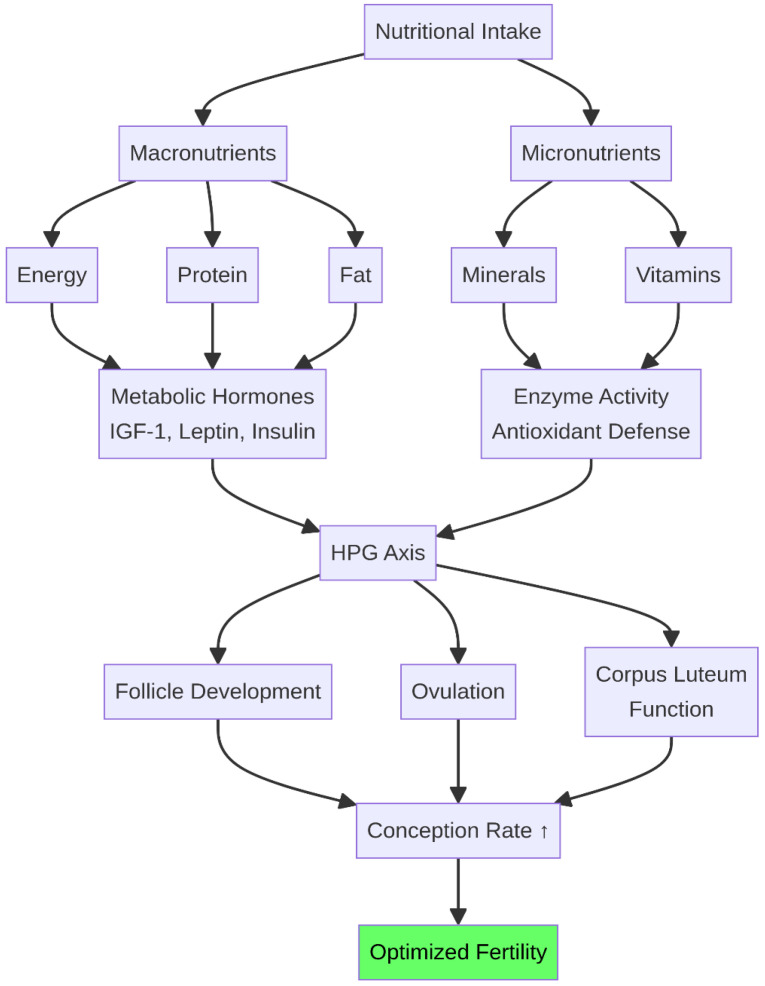
Comprehensive nutritional regulation of reproduction. This integrative diagram illustrates how macronutrients and micronutrients regulate metabolic hormones and antioxidant defense systems, which in turn control the HPG axis and key reproductive events to optimize fertility in ruminants. ↑ indicates an increase.

The relationship between nutrition and reproduction in ruminants is a complex, multifactorial system where systemic energy balance, macronutrient composition, and micronutrient availability converge to regulate endocrine signaling and cellular function (Figure 9). The physiological conflict between high production and fertility necessitates a deep, mechanistic understanding of these interactions to develop effective nutritional interventions. Strategies must move beyond simply meeting basic requirements and focus on providing specific nutrients at critical physiological stages to modulate key reproductive pathways.

Future research should increasingly focus on the fields of nutrigenomics and metabolomics to elucidate how individual genetic variation influences an animal’s response to specific dietary components. The emerging understanding of the kisspeptin–KNDy neuron system as the central mediator between metabolic status and GnRH secretion opens new avenues for targeted interventions. Furthermore, a greater understanding of the mechanisms of fetal programming and its epigenetic basis will allow nutritional interventions in one generation to enhance the reproductive fitness of the next. The integration of precision livestock farming technologies (including real-time metabolic monitoring and individual animal data management) with these advanced scientific insights will enable the development of truly personalized and adaptive feeding strategies. By combining these approaches, it will be possible to develop sustainable management systems that optimize both the productivity and reproductive health of high-yielding ruminants, ensuring long-term herd efficiency and welfare.

## Figures and Tables

**Table 1:** Summary of key micronutrients, their primary reproductive functions, deficiency consequences, and recommended supply ranges for ruminants.

Nutrient	Primary Reproductive Function	Deficiency Consequences	Recommended Supply Range
Selenium (Se)	Cofactor for glutathione peroxidase (GSH-Px); antioxidant defense for gametes and embryos	Retained fetal membranes, metritis, increased embryonic loss, white muscle disease in offspring	0.1–0.3 mg/kg DM
Copper (Cu)	SOD cofactor; catecholamine metabolism; GnRH secretion	Delayed puberty, anovulation, immune dysfunction	10–20 mg/kg DM
Zinc (Zn)	SOD cofactor; steroid hormone synthesis; GnRH regulation; sperm integrity	Impaired steroidogenesis, reduced conception rate, fetal malformations	40–80 mg/kg DM
Manganese (Mn)	Cofactor for cholesterol/steroid synthesis; progesterone and estrogen production	Irregular estrous cycles, low conception rates, skeletal malformations in offspring	20–40 mg/kg DM
Vitamin A/β-Carotene	Uterine epithelial integrity; nuclear receptor-mediated gene expression in reproductive tissues; oocyte maturation	Impaired implantation, night blindness, increased embryonic loss	Vit A: 2200–3300 IU/kg DM; β-Carotene: 300–400 mg/cow/day
Vitamin D	VDR-mediated regulation of folliculogenesis and steroidogenesis in granulosa cells	Disrupted follicular development, subfertility, skeletal disorders	1000–2000 IU/kg DM
Vitamin E	Synergistic antioxidant with Se; protects gametes and uterine tissue from lipid peroxidation	Retained placenta, uterine infections, impaired immune function peripartum	500–1000 IU/cow/day (transition period)
Cobalt (Co)/Vit B12	Methionine synthase cofactor; one-carbon metabolism; fetal development	Impaired fetal growth, anemia, reduced reproductive efficiency	Co: 0.11–0.20 mg/kg DM

DM: dry matter. Values represent general guidelines; actual requirements vary by species, production stage, and diet composition. Data compiled from references [18,19,20,21,22,23,24,25,26,27,36,37,38,39].

## Data Availability

No new data were created or analyzed in this study. Data sharing is not applicable to this article.

## Abbreviations

ALA	α-linolenic acid
ARC	Arcuate nucleus
BCS	Body condition score
BHB	Beta-hydroxybutyrate
CL	Corpus luteum
Co	Cobalt
Cu	Copper
DHA	Docosahexaenoic acid
DM	Dry matter
EPA	Eicosapentaenoic acid
GH	Growth hormone
GSH-Px	Glutathione peroxidase
GnRH	Gonadotropin-releasing hormone
HPG	Hypothalamic–pituitary–gonadal
IGF-1	Insulin-like growth factor-1
LH	Luteinizing hormone
Mn	Manganese
NEB	Negative energy balance
NEFA	Non-esterified fatty acids
NO	Nitric oxide
NOS	Nitric oxide synthase
PGF2α	Prostaglandin F2α
PUFAs	Polyunsaturated fatty acids
RAR/RXR	Retinoic acid receptors
RDP	Rumen-degradable protein
ROS	Reactive oxygen species
SOD	Superoxide dismutase
Se	Selenium
VDR	Vitamin D receptors
PHGPx/GPx5	Phospholipid hydroperoxide glutathione peroxidase
NASEM	National Academies of Sciences, Engineering, and Medicine
MUN	Milk urea nitrogen
KNDy	Kisspeptin-neurokinin B-dynorphin
FSH	Follicle-stimulating hormone
BUN	Blood urea nitrogen
Zn	Zinc
